# The Growing Price Gap between More and Less Healthy Foods: Analysis of a Novel Longitudinal UK Dataset

**DOI:** 10.1371/journal.pone.0109343

**Published:** 2014-10-08

**Authors:** Nicholas R. V. Jones, Annalijn I. Conklin, Marc Suhrcke, Pablo Monsivais

**Affiliations:** 1 UK Clinical Research Collaboration (UKCRC) Centre for Diet and Activity Research, Department of MRC Epidemiology, University of Cambridge School of Clinical Medicine, Addenbrooke's Treatment Centre, Cambridge Biomedical Campus, Cambridge, United Kingdom; 2 Faculty of Medicine and Health Sciences, University of East Anglia, Norwich, United Kingdom; Old Dominion University, United States of America

## Abstract

**Objectives:**

The UK government has noted the public health importance of food prices and the affordability of a healthy diet. Yet, methods for tracking change over time have not been established. We aimed to investigate the prices of more and less healthy foods over time using existing government data on national food prices and nutrition content.

**Methods:**

We linked economic data for 94 foods and beverages in the UK Consumer Price Index to food and nutrient data from the UK Department of Health's National Diet and Nutrition Survey, producing a novel dataset across the period 2002–2012. Each item was assigned to a food group and also categorised as either “more healthy” or “less healthy” using a nutrient profiling model developed by the Food Standards Agency. We tested statistical significance using a t-test and repeated measures ANOVA.

**Results:**

The mean (standard deviation) 2012 price/1000 kcal was £2.50 (0.29) for less healthy items and £7.49 (1.27) for more healthy items. The ANOVA results confirmed that all prices had risen over the period 2002–2012, but more healthy items rose faster than less healthy ones in absolute terms:£0.17 compared to £0.07/1000 kcal per year on average for more and less healthy items, respectively (p<0.001).

**Conclusions:**

Since 2002, more healthy foods and beverages have been consistently more expensive than less healthy ones, with a growing gap between them. This trend is likely to make healthier diets less affordable over time, which may have implications for individual food security and population health, and it may exacerbate social inequalities in health. The novel data linkage employed here could be used as the basis for routine food price monitoring to inform public health policy.

## Introduction

The association between foods, nutrients and diets and certain health outcomes is well established, [1] with the World Health Organization identifying energy-dense, nutrient-poor foods that are high in fat, sugar and salt as contributing to excess risk of chronic disease. [2] Accordingly, dietary recommendations, including those from the UK Department of Health (DH), discourage the consumption of such foods and emphasise vegetables and fruits, whole grains, low fat dairy foods and lean sources of protein. [3] However, on average the UK population consumes an excess of saturated fat and non-milk extrinsic sugars, and fails to consume enough oily fish or fruit and vegetables, falling short of government recommendations. [4] These patterns have a marked effect on public health: the 2010 Global Burden of Disease Study found that unhealthy diets accounted for 14.3% of the UK's disease burden (measured in disability-adjusted life years). [5] The burden on the healthcare system is also considerable with diet-related ill health estimated to be responsible for £5.8 billion of National Health Service expenditure annually, more than either smoking, alcohol consumption or physical inactivity (based upon data from 2006–7). [6].

One factor that might limit the uptake of healthier diets is the cost of healthier foods, which has not been recognised by the majority of UK public health policy outside of the context of national food security. [7], [8] The notion that healthier foods are more expensive and that this expense contributes to the consumption of unhealthy diets is not new, and has a strong evidence base and conceptual framework to support it. [9] In a 2013 survey, price was rated by UK consumers as the most important factor influencing their choices of food products, with 39% stating it was the factor of greatest importance and 91% listing it in their top five criteria. [10] In contrast, just 9% of UK consumers considered a food's healthiness to be the most important factor and only 49% placed it in the top five. [10] These findings suggest that cost considerations may override health concerns in consumer dietary choices.

Both researchers and policymakers have recognised that the price of healthy diets and foods ought to be monitored to inform public health nutrition policy but no monitoring system has hitherto been established in the UK. [8], [11] Here we outline a method for examining the cost of foods in relation to nutrient content by linking existing UK government economic and nutrition surveillance data. Using the resulting dataset, we then examine changes in the price of food between 2002 and 2012, by Eatwell food group and by category of healthfulness as determined by a widely used nutrient profiling score. Our approach to linking such existing data could provide the basis for routine monitoring of the affordability of healthy foods and diets, thereby allowing for a better understanding of how price differs between more or less healthy foods.

## Methods

### Ethics Statement

NDNS was conducted according to the guidelines laid down in the Declaration of Helsinki and all procedures involving human subjects were approved by the Oxfordshire A Research Ethics Committee. Written informed consent was obtained from all participants [12].

### Methods summary

We obtained food price and nutrition data from two separate and publicly available sources and linked the two together to create a novel dataset. We then converted prices to a price-per-unit-of-energy value and classified food items according to their nutritional content and by food group. Finally, we compared the mean prices of these categories in 2012 and examined the change in price since 2002.

### Food price data

We selected the foods and beverages in our sample from the list of goods and services used to calculate the Consumer Price Index (CPI), a tool used to measure inflation in the UK based upon a basket of goods for which prices are measured across the nation each quarter. [13] In 2012, this basket included 157 foods and beverages which did not include an element of service, for example, a hot meal in a pub. We excluded such items because the cost of the service could not be separated from the cost of the food. However, to ensure that a meaningful comparison was being made over time, we restricted the contents of the basket analysed to include only those goods that remained in the basket during our study period between 2002 and 2012. Research in the USA has indicated that market baskets that change over time can contribute to apparent differences in the rate of change in the price of food groups. [14] We further excluded an additional four items in the basket as they contained no nutrients meaningful to the research questions (Instant Coffee, Filter Coffee, Tea Bags, Bottled Mineral Water), leaving a final list of 94 foods and beverages for comparison across 10 years.

We took the median price for each good in a given quarter and produced a mean value for the year, using data taken from the Office for National Statistics. [15] These data were per-unit prices, with no separate field for unit weight. To establish the price per 100 g, we used information on the purchased weight where it was included in the food name (e.g. “Grapes per kg”), or inferred the purchased mass using information on similar items available for purchase on an online supermarket aggregator, *mySupermarket*. [16] We chose items from the site when the price was closest to the 2012 price taken from the CPI data. For items with variable weights, e.g. peaches, we assigned the weight recorded for that type of item in the USDA National Nutrient Database for Standard Reference. [17] Using this combined method we established a price per 100 g for all 94 items in our list.

### Nutrition data

We obtained nutrition data from survey years 1–3 of the rolling programme of the nationally-representative National Diet and Nutrition Survey (NDNS), which was available from the Economic and Social Data Service. [18] Foods consumed by 1,491 adult survey respondents have their nutrient content reported in detail, with the content of 60 nutrients and 27 disaggregated foods (e.g. mass of dried fruit) per portion and total portion mass reported. We removed non-nutrient information from the Food Level Dietary Data file, removed duplicate entries, and converted the values to a 100 g scale, thus creating a database of 3790 unique foods and beverages.

### Linkage process

We used a qualitative process to determine the most appropriate NDNS items for each of the 94 items from the CPI list. Where multiple NDNS items were deemed a suitable match for a CPI item we calculated mean nutrition data, weighted by the frequency of consumption for each NDNS item. We adopted this approach to account for the fact that some CPI items were only described in very broad terms and could cover a range of different foods (e.g. “frozen ready meal, cooked – serves 1”), and also to account for the range of different methods of preparation (for instance, a potato could be boiled, baked or fried) which were associated with NDNS food items and would alter the nutrient content. The most NDNS items matched to any one item was 14 and the least was 1, with a median of 2 NDNS items match for a given CPI item.

Once matches had been made, the nutrition data needed to be adjusted to account for the fact that the NDNS data concern foods as consumed and the CPI data foods as purchased. The incongruence in the data occurs because there are either losses or gains associated with preparation and cooking of many of the listed foods. To remedy this we adjusted the CPI food price data so that they expressed price in terms of price per 100 g edible portion, using edible portion figures from the *United States Department of Agriculture's Handbook 102* where necessary. [19].

Following this adjustment, we converted prices per 100 g into price per 1000 kcal using the data on energy content provided by the NDNS. We produced a dataset with detailed nutrient content per 100 g and 11 years of data on price per 1000 kcal which would allow us to analyse price changes over time in relation to nutrient content.

### Food group-based classification of foods

We classified food items in our new dataset according to five distinct food groups, defined by the Eatwell Plate—a tool for nutrition communication developed by the DH to define a healthy diet. [3] The five groups analysed were: (i) bread, rice potatoes and pasta; (ii) fruit and vegetables; (iii) milk and dairy foods; (iv) meat, fish, eggs, beans and other sources of protein; and (v) food and drinks high in fat and/or sugar. We assigned foods to food groups using a reference table in the Livewell Report which matched NDNS food categories to Eatwell food groups. [20] When NDNS items corresponding to a CPI item included more than one food group, we applied only one food group based on the item most frequently consumed according to the NDNS survey data.

### Nutrient-based classification of foods

Food items were also classified as “more healthy” or “less healthy” based upon nutrient profiling, a technique which accounts for a food or drink's overall nutritional characteristics. We used the WXYfm model detailed in the DH's Nutritional Profiling Technical Guide, hereafter referred to as the “FSA score” due to its historical usage by the Food Standards Agency (FSA), because this score provides a categorical distinction between more and less healthy foods. [21] This model assigns an overall numeric score according to per 100 g levels of: energy, saturated fat, total sugar, sodium, fibre, protein, and fruit, vegetable and nut content. This particular model was originally developed to highlight foods which should not be advertised to children and consequently gives a definition of less healthy foods, allowing for objective classification of the items in our sample. [21] When used to classify foods this score has been shown to have good agreement with the subjective opinions of nutrition professionals. [22].

### Statistical analyses

Descriptive statistics were used to characterise the mean economic cost and change in cost over time, according to food group and FSA score healthfulness categories. The distributions of the nutrient profile score for each Eatwell food group were tested with ANOVA using the data for 2012. The difference in price in 2012 between the categories “less healthy” and “more healthy” was tested using a t-test and between the Eatwell groups with ANOVA. Repeated measures ANOVA were used to determine whether (a) there was a statistically significant change in price over the period 2002–2012 for all foods and (b) the prices changed differently between Eatwell food group and FSA categories over this period.

Analyses were conducted using Stata (version SE 12.1). [23] Figures were produced using *R* (version 2.15.1 for *Windows*) and the *ggplot2* package. [24], [25].

### Sensitivity Analyses

We conducted two sensitivity analyses: we analysed the difference in price between more and less healthy foods with fruit and vegetables removed from the healthy foods category, to test whether any price difference was due only to this group. We also tested for the possibility of bias caused by excluding items which did not appear in all years, by looking at the difference in price between more and less healthy foods when all items in 2012 were included, not just those which appeared in the CPI basket across the entire 2002–12 period.

## Results

**Table 1** shows the 2012 mean price and changes in price for 2002–2012, 2002–2007 and 2007–2012, in both absolute and relative terms, for all foods and by Eatwell and FSA score categories. Between 2002 and 2012 the mean price of all foods in our sample rose 35%, from £3.87/1000 kcal to £5.21/1000 kcal. This increase was not constant, with the prices rising at a greater rate after 2007 than before, with the absolute change for the period 2002–07 being £0.27/1000 kcal compared to £1.07/1000 kcal for the period 2007–12. This increase in the rate of change after 2007 applied to all food groups and nutrient composition categories.

**Table 1:** 2012 mean, absolute and relative changes in price for all foods, by Eatwell group and by FSA score category.

		*All foods*	*Bread, rice, potatoes, pasta*	*Fruit and vegetables*	*Milk and dairy foods*	*Meat, fish, eggs, beans, other sources of protein*	*Food & drinks high in fat and/or sugar*	*More healthy*	*Less healthy*
*2012 mean price (£/1000 kcal)*		*5.21*	*1.26*	*9.13*	*4.75*	*4.93*	*3.11*	*7.49*	*2.50*
*Relative change in price (%)*	*2002*–*12*	*35*	*12*	*23*	*29*	*54*	*49*	*33*	*41*
	*2002*–*07*	*7*	*-9*	*4*	*9*	*11*	*11*	*7*	*8*
	*2007*–*12*	*26*	*22*	*18*	*18*	*39*	*33*	*24*	*31*
*Absolute change in price (£/1000 kcal)*	2002–12	*1.34*	*0.13*	*1.73*	*1.07*	*1.73*	*1.02*	*1.84*	*0.73*
	2002–07	*0.27*	*−0.10*	*0.32*	*0.34*	*0.34*	*0.24*	*0.37*	*0.14*
	2007–12	*1.07*	*0.23*	*1.41*	*0.73*	*1.39*	*0.78*	*1.47*	*0.59*

### Price differences by Eatwell food group

**Figure 1:**
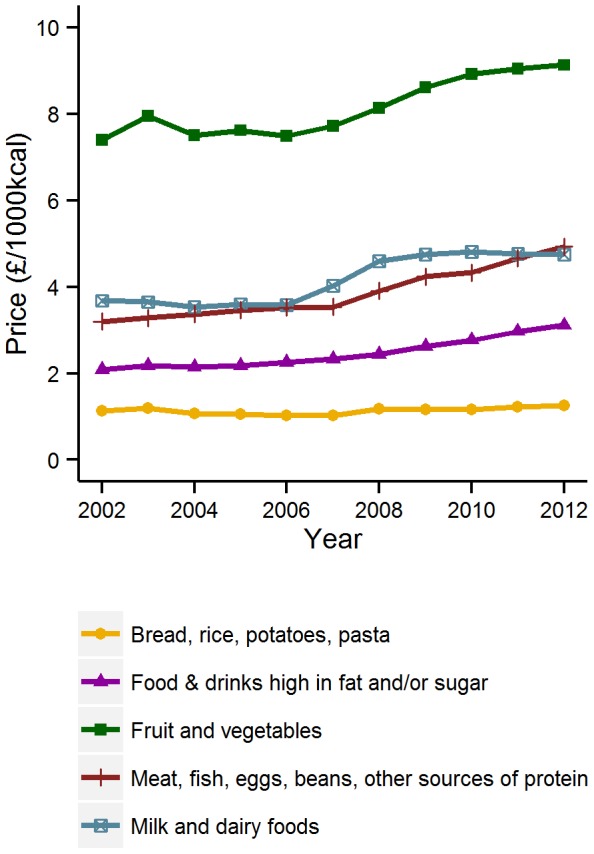
Mean price of foods by Eatwell food group, 2002–2012. Mean price (£/1000 kcal) by Eatwell food group of foods and beverages remaining in the UK Consumer Price Index basket across the entirety of the period 2002–2012 (n = 94).

The mean prices per 1000 kcal for each of the Eatwell food groups for the period 2002–2012 are shown in **Figure 1**. The figure shows that there is a clear hierarchy of prices across the period in which fruit and vegetables are always the most expensive foods and starchy carbohydrates the least expensive. All food groups saw price increases over the observation period, except for starchy foods, whose average price remained broadly constant throughout. The difference between groups was significant across all years (p<0.001).

### Comparison of food groups' nutrient composition

**Figure 2:**
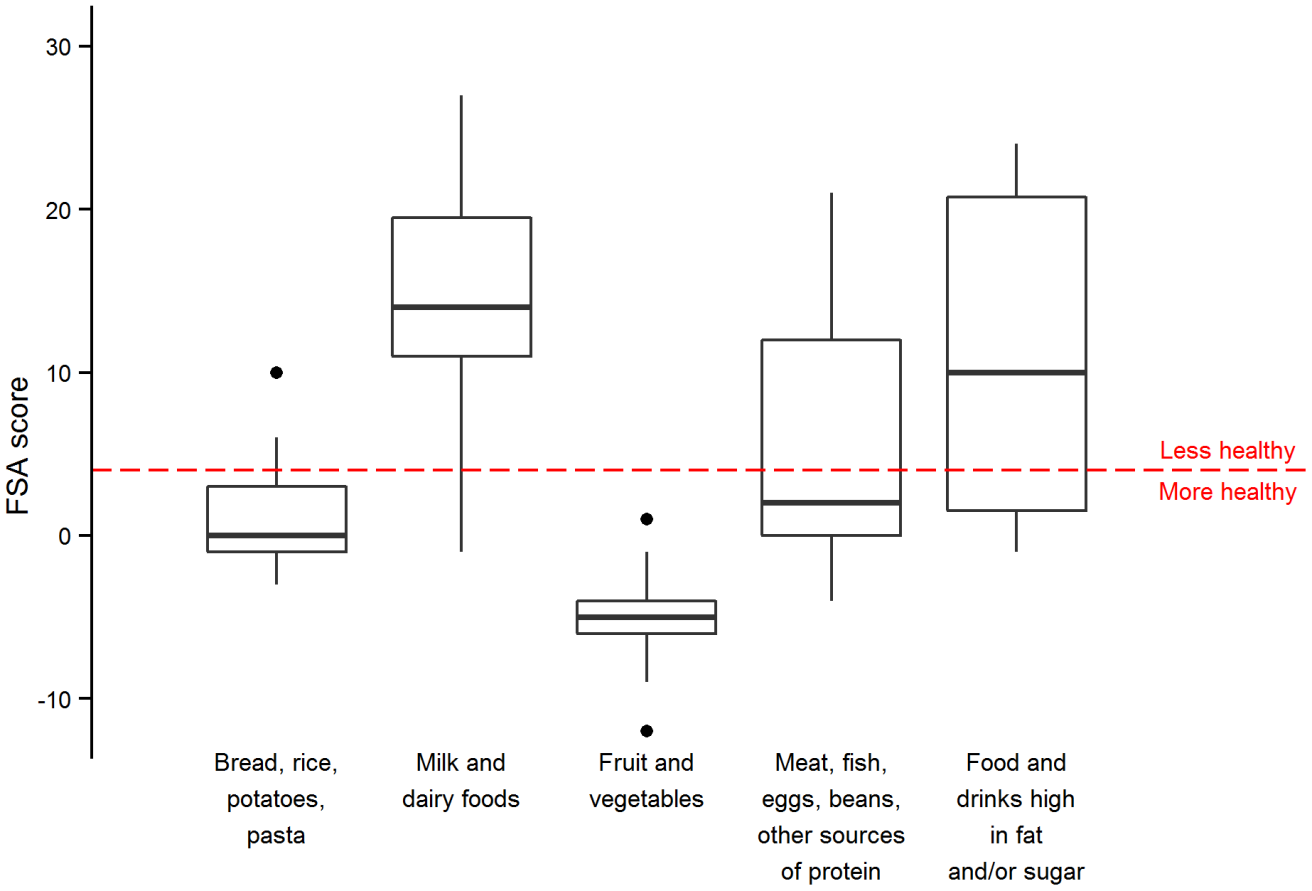
Box plots of nutrient density by Eatwell food group. Box plots of nutrient density as defined by the Food Standards Agency nutrient profiling score for foods (and not beverages) remaining in the UK Consumer Price Index basket across the entirety of the period 2002–2012 (n = 88), by Eatwell food group.

**Figure 2** shows the distribution of nutritional value for each Eatwell group according to the FSA score. We found that only the fruits and vegetables group showed a distinct, healthier nutrient profile, while all other groups contained food items overlapping the Ofcom categories of more and less healthy (for visual clarity, this plot excludes the sample's six beverages sample because the score uses different cut-offs for foods and beverages. All other analyses include all 94 foods and beverages). When tested with an ANOVA, there was a significant difference between FSA scores by groups (p<0.001) but this was not the case when the “Fruit and Vegetables” category was removed (p = 0.267). This result indicates that with the exception of this category, a food's Eatwell group cannot be used to determine whether a food is more or less healthy. This plot excludes the six beverages in the sample because the score uses a separate cut-off for foods and beverages. The beverages are included for all other analyses.

### Price differences by nutrient profile category

**Figure 3:**
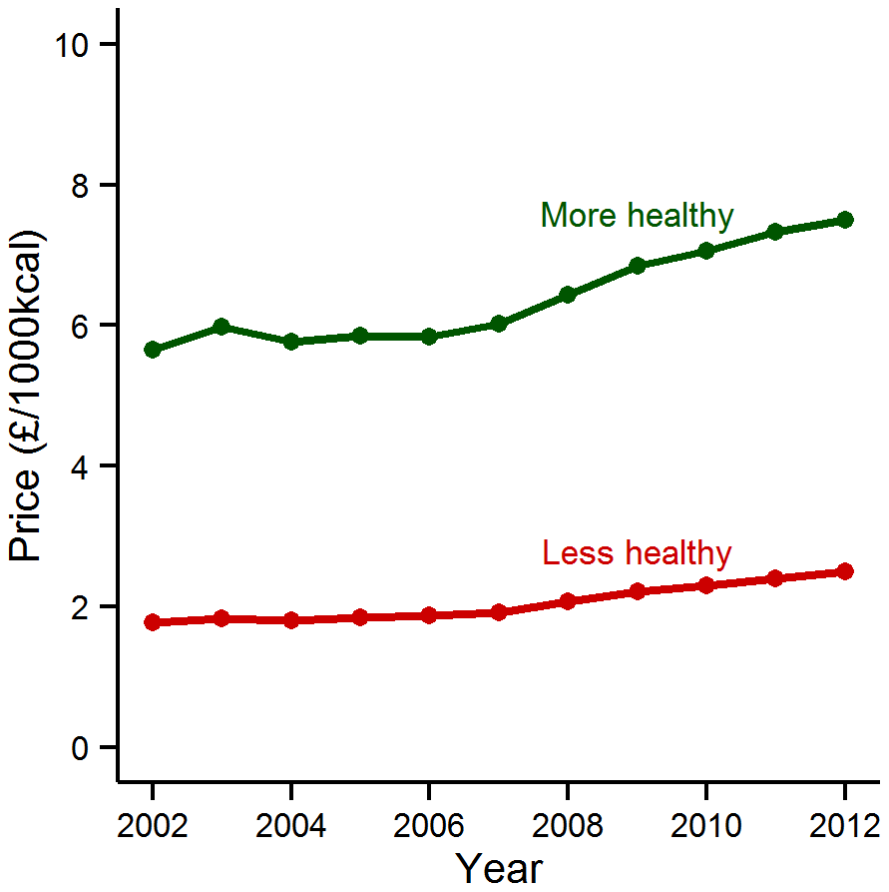
Mean price of foods by Food Standards Agency nutrient profiling score category, 2002–2012. Mean price (£/1000 kcal) by Food Standards Agency nutrient profiling score category of foods and beverages remaining in the UK Consumer Price Index basket across the entirety of the period 2002-2012 (n = 94).

We found an absolute difference in price between the nutrient profile categories in 2012, with more healthy foods approximately three times more expensive than less healthy foods (p<0.001). The mean price was £2.50 (standard deviation  = 0.29) for less healthy items and £7.49 (1.27) for more healthy items. The mean prices per 1000 kcal for foods categorised as less healthy and foods categorised as more healthy for the period 2002–2012 are shown in **Figure 3**. Across the study period, we found that there was a consistent difference in price between these groups (p<0.001) and that there was a difference in the change over time by group (p = 0.008).

### Sensitivity Analyses

When we included all foods and beverages in the CPI in 2012 rather than only those which has been included in the CPI basket since 2002, the difference in mean price between more and less healthy foods was £6.57/1000 kcal and £2.96/1000 kcal respectively (p<0.001). We also tested for a difference between more and less healthy foods over time when fruit and vegetables were removed from the analysis, finding that the difference between groups remained (p<0.001) and that they showed different time trends (p = 0.004), with the price of more healthy foods rising by £1.95/1000 kcal over the period in comparison to £0.73/1000 kcal for less healthy foods. With fruit and vegetables excluded, the mean price for more healthy foods in 2012 was £5.78/1000 kcal whereas the mean price of less healthy foods did not change.

## Discussion

Our results show that the price of more healthy foods was consistently greater than that of less healthy foods over the period 2002–2012, and that the absolute price gap between healthy and less healthy foods has grown over this period. This finding strengthens the case for monitoring the affordability of healthy foods and diets in order to inform potential economic policy responses.

This study is the first to use UK data to examine price trends by the nutrient composition of foods. Our results tally with the general trend of increasing food prices observed in similar high income nations, as reported in a review, [26] where studies have found that in recent years healthy foods had increased more in price than foods which were less healthy, [27]–[30] and that healthier versions of particular foods were more expensive. [31], [32] Another recent review has again found that within given food groups, the healthier option was typically more expensive for meats/protein, snacks/sweets, grains, and fats/oils, whilst healthier dairy foods were found to be less expensive. [33].

The broadly consistent observation of disparities in food prices across countries is likely to have multiple causes. In the UK it may be the case that food prices are heavily influenced by certain features of the Common Agricultural Policy (CAP) which intervenes in food markets to subsidise the production of certain goods, including grains, dairy products, oils and sugar. [34] Such subsidies have the potential to affect public health by influencing the availability and price of foods, with modelling studies showing that the CAP's presence may reduce the quantity of fruit and vegetables consumed and increase cardiovascular mortality through encouraging the consumption of saturated fats. [35], [36] If public health policy is required to address the issue of the higher price of more healthy foods, it is likely to be necessary to engage with supply-side issues such as CAP reform to achieve long-term change.

In recent years the issue of food poverty has been of increasing concern in the UK and the rising use of food banks has been recognised as an issue of public health importance. [37], [38] Our results suggest that we should consider not only the issue of people being able to afford to eat enough food to avoid hunger but also being able to eat enough food which is healthy. The standard definition of food security is that people should have physical and economic access to “sufficient, nutritionally adequate and safe food”, [39], [40] meaning that if economic constraints are gradually forcing people to replace more healthy foods with less healthy ones, they are becoming increasingly exposed to the risk of food insecurity. Analyses of UK food spending data by the Institute for Fiscal Studies has shown that, in recent years, all SES groups have changed their purchasing habits to both spend less on food and purchase calories which are both cheaper and less healthy, for example purchasing less fruit but more grains, cheese and prepared dishes. [41] Our findings help to explain this observation by uncovering the magnitude of the price difference between more and less healthy foods, which is a factor that drives increasing food insecurity and could contribute to a deterioration in population health.

In addition to our main finding we also observed that, with the exception of “fruit and vegetables”, the Eatwell food groups were not distinct in terms of the FSA scores of their constituent foods. This finding is consistent with earlier work in the US showing that food groups have limited value in classifying healthy and less healthy foods. [42] If monitoring of food prices is to start in the UK, we would echo these authors in arguing for the development of a monitoring tool that merges nutritional profiling techniques with current guidance for eating a balanced diet rather than relying on monitoring prices by food group alone.

### Methodological considerations and limitations

A key weakness to acknowledge is that the number of foods and beverages included in this study is small and only reflects those foods included in the CPI rather than a full range of available foods. Nevertheless, the items included for analysis should reflect those most commonly purchased by UK consumers, given the CPI's role as a measure of consumer inflation. Our study also does not account for variation in price by region or outlet, which is likely to affect the absolute and relative difference in price between food types in given instances. Another potential weakness is the use of price per unit energy rather than price per unit mass or any other price denominator, given that one of the factors determining a food's health categorisation is its energy content, an issue which has been raised by others. [33], [43] We analysed price per unit of energy in line with the approach used by international organisations to assess food poverty. [44], [45] Moreover, price per unit energy is more consistent with dietary guidance than price per unit weight and with observed household purchasing behaviour which shows that energy consumption is broadly consistent in the UK, even across differing SES groups. [46], [47].

The main strength of our study is that we demonstrate the utility of existing government datasets on the prices and nutrition content of foods purchased and consumed in the UK, addressing the recent call by the International Network for Food and Obesity/Non-communicable Diseases Research, Monitoring and Action Support (INFORMAS) for a cost-effective and simple tool for monitoring the price of healthy foods. [11] The study also provides information on recent price trends pertinent to the health of the UK population, using an objective and nutritionally relevant tool to categorise the foods and beverages examined.

### Unanswered questions and future research

In conjunction with other work in the area of food prices, food poverty and food security, our findings highlight the need for the routine monitoring of food prices in relation to the food's nutrient composition. For greater relevance these prices should also be considered in the context of income and other unavoidable expenditure, such a rent or utilities, making it possible to consider the affordability of a healthy diet. Future research in this area ought to build on our findings and examine the cost of observed diets in relation to their quality, since the quality and cost of the overall diet is not simply a function of the price of certain healthy foods being more expensive. Future research should also seek to go beyond a descriptive analysis of the price trends by themselves and try to assess the cause of the observed link between the price and healthiness of foods, and also to examine the link between food purchases, actual consumption and health outcomes.

## Conclusions

We have demonstrated a novel linkage of existing economic and nutrition surveillance data to assess trends in the prices of foods in relation to their nutritional value. The growing gap in the price of more healthy and less healthy foods revealed by our analysis leads us to suggest that ongoing monitoring of food prices for public health is warranted. The data linkage we describe could underpin such food price monitoring and provide evidence to inform policy responses to the problem of rising food prices.
